# Multiplex immunohistochemistry for immune profiling of HPV-associated head and neck cancer

**DOI:** 10.1186/2051-1426-3-S2-P419

**Published:** 2015-11-04

**Authors:** Takahiro Tsujikawa, Rohan N Borkar, Vahid Azimi, El Edward Rassi, Daniel R Clayburgh, Sushil Kumar, Andrew J Gunderson, Molly F Kresz-Martin, Paul W Flint, Lisa M Coussens

**Affiliations:** 1 Oregon Health & Science University, Portland, OR, USA; 2 Intel Corporation, Hillsboro, OR, USA

## 

Human Papilloma Virus (HPV)-positive head and neck squamous cell carcinoma (HNSCC) is clinically distinct from HPV-negative HNSCC, and as such requires differential therapeutic approaches. Accumulating evidence indicates a significant linkage between the immune response within the tissue and pathogenesis of HPV-associated HNSCC. To further elucidate immune-related signatures in HPV-associated HNSCC, we performed multiplex histological analysis in de-identified tissue microarray sections including HPV-positive (n = 21), HPV-negative (n = 17), and normal oropharynx (n = 8). Following immunohistochemistry (IHC) for CD45, CD3, CD8, Foxp3, T-bet, GATA-3, RORgT, CD20, CD56, CD68, MHC class II, CSF1R, CD66b, tryptase, CD83, DC-SIGN, PD-1, and PD-L1, the cell intensity per mm^2^ ratio/composition, localization were quantitatively evaluated. The HPV-status was confirmed by HPV16/18 polymerase chain reaction and IHC for p16^INK4a^. IHC for p16 or EpCAM were utilized for defining tumor region. Infiltration of T cell populations including CD45^+^CD3^+^CD8^+^ T cells (*P* < 0.01), CD45^+^CD3^+^CD8^−^Foxp3^+^ regulatory T cells (*P* < 0.05) and CD45^+^CD3^+^CD8^−^Foxp3^+^T-bet^+^ Th1 cells (*P* < 0.01), CD45^+^CD20^+^CD3^−^B cells (*P* < 0.05), CD45^+^CD68^+^CD163^−^ macrophages (*P* < 0.001), and CD45^+^Tryptase^+^ mast cells (*P* < 0.01) was significantly higher in the HPV-positive group than in the HPV-negative group. CD8/CD68 ratio of HPV-positive tumor was higher than that of HPV-negative tumor (*P* < 0.0*5*), and the highest CD163^−^CD68^+^/CD163^+^CD68^+^ ratio was observed in the intra-tumor region of HPV-positive tumors. High PD-L1 expression on CD68^+^CD163^+^ macrophages and MHC class II^+^CD83^+^ dendritic cells was intensively observed in the intra-tumor region of the HPV-positive group while maturation of dendritic cells assessed by CD83/DC-SIGN ratio was significantly higher in the peritumoral stroma than intra-tumor regions (*P* < 0.05), indicating distinct immune regulatory mechanisms between intra and peritumoral regions. These signatures provide further evidence of anti-tumor immunity against HPV-positive head and neck cancer, and potentially lead to personalized treatment including immunomodulatory therapeutic targets specialized for the HPV/immune status.

